# Therapeutic Potential of Seaweed-Derived Laminaran: Attenuation of Clinical Drug Cytotoxicity and Reactive Oxygen Species Scavenging

**DOI:** 10.3390/antiox12071328

**Published:** 2023-06-23

**Authors:** Hiromi Kurokawa, Thomas Kiran Marella, Hirofumi Matsui, Yutaka Kuroki, Makoto M. Watanabe

**Affiliations:** 1 Algae Biomass Energy System R&D Center, University of Tsukuba, Tennodai 1-1-1, Tsukuba 3058572, Japan; tomccmb@gmail.com (T.K.M.); hmatsui@md.tsukuba.ac.jp (H.M.); mwatanabe@algae-consortium.jp (M.M.W.); 2 Faculty of Medicine, University of Tsukuba, Tennodai 1-1-1, Tsukuba 3058575, Japan; 3 Delightex Pte. Ltd., 230 Victoria Street, #15-01 Bugis, Junction Towers, Singapore 188024, Singapore; yutaka.kuroki@jt.com

**Keywords:** laminaran, seaweed, reactive oxygen species, antioxidant, electron spin resonance

## Abstract

β-glucan has been shown to be effective for several diseases such as immune regulation and blood pressure suppression. Seaweed contains a β-1,3/1,6-glucan called laminaran. The present commercial source of β-glucan is black yeast; however, a fermentation process using organic carbon substrates makes production unsustainable, whereas macroalgae provide a sustainable alternative with the use of CO_2_ and seawater as growth substrates. However, bioactivity studies on laminaran are limited. We aimed to evaluate whether laminaran can scavenge reactive oxygen species (ROS) and attenuate cytotoxicity caused by clinical drugs such as indomethacin (Ind) and dabigatran (Dab). Electron spin resonance assay revealed that laminaran scavenged singlet oxygen (^1^O_2_) and superoxide anions (O_2_^•^^−^) directly but did not scavenge hydroxyl radicals (^•^OH). Mitochondrial ROS detection dye showed that laminaran scavenged mitochondrial O_2_^•^^−^ produced upon administration of Ind or Dab. Moreover, significant reductions in ^•^OH and peroxynitrate (ONOO^−^) levels were observed. Since ^•^OH and ONOO^−^ are generated from O_2_^•^^−^ in the cells, laminaran could indirectly suppress the generation of ^•^OH and ONOO^−^ via the removal of O_2_^•^^−^. Both Ind and Dab induce cell injury via ROS production. Laminaran attenuated the cytotoxicity derived from these drugs and may represent a functional food with anti-aging and disease prevention properties.

## 1. Introduction

Noncommunicable diseases (NCDs) account for 40 million deaths annually, which represent approximately 70% of all deaths globally. The key causes of NCDs include physiology, genetics, environmental factors, and lifestyle choices including dietary preferences. Diabetes, chronic kidney disease, and cardiovascular disease are the major causes for most deaths attributed to NCDs. One of the key causes of these diseases is behavioral risk factors, which include poor nutrition and decreased dietary fiber intake [1,2]. Increased fiber intake significantly reduces the risk of hypertension, stroke, coronary heart disease, certain gastrointestinal disorders, diabetes, and obesity. Dietary fiber intake can improve blood glucose levels and insulin sensitivity in patients with diabetes; facilitate weight loss in individuals with obesity; and alleviate gastroesophageal ulcers, constipation, and hemorrhoids related to gastrointestinal diseases [3,4,5]. β-glucans are key dietary fibers composed of β-D-glucose polysaccharides and are found in the cell walls of bacteria, fungi, and algae. β-glucan in different structural forms can be obtained from different natural resources, such as water-insoluble β-1,3-glucan derived from the bacterium *Alcaligenes*, alga *Euglena*, fungus *Polia*, and *Grape vitis*; water-soluble β-1,3/1,4-glucan from cereal, Iceland moss, and oyster mushroom; and β-1,3/1,6-glucan from seaweeds, molds, mushrooms, and yeasts [6]. In particular, the latter two water-soluble glucans have been used as health supplements and food additives with the approval of the government or its agencies. β-1,3/1,4-glucan consumption is known to reduce blood glucose levels and modulate the gut microbiome [7]. Food supplements containing β-1,3/1,4-glucan that reduce plasma cholesterol levels and the risk of heart disease were approved by the Food and Drug Administration in 1997 [8].

Compared to cereals and other plant sources, the production of β-1,3/1,6-glucans from unicellular organisms such as yeast and bacteria is increasingly being explored owing to the availability of scale-up processes and related downstream processing systems. A major producer of β-1,3/1,6-glucan is the black yeast *Aureobasidium pullulans*, which releases glucan as an extracellular exudate [9,10]. β-1,3/1,6-glucan was approved for use as a food additive by the Ministry of Health of the Japanese Government in 1996. It displays several health benefits, such as anti-inflammatory and anti-stress activities, atherosclerosis reduction, immune system upregulation, and hyperinflammation downregulation [11,12,13]. The effect of *A. pullulans*-derived glucan on the cytokine storm and coagulopathy in patients with coronavirus disease 2019 (COVID-19) was evaluated in a randomized pilot clinical study. Supplementation with this glucan was found to help regulate the major biomarkers of clinical severity and mortality in COVID-19, including interleukin-6, D-dimer, and neutrophil-to-lymphocyte ratio over 15 and 30 d compared with those in patients who underwent standard care alone.

Apart from black yeast, marine seaweed represents an alternative natural source of β1,3/1,6-glucan, known as laminaran, which is stored in brown algae. *Laminaria digitata*, a commonly available wild seaweed species, may contain >50% laminaran by weight [14]. This amount of laminaran is almost the same as that in black yeast. Seaweed has been consumed as food and condiments in East Asia since ancient times. Black yeast-derived glucan is produced using a fermentation tank system with organic carbon sources as carbon substrates, which results in CO_2_ emissions. In contrast, laminaran has the advantage that it is synthesized through absorbing CO_2_ via photosynthesis in wild seaweeds and can be consumed directly even without further downstream processing. However, studies on the bioactivity of laminaran are limited to its antitumor, anti-inflammatory, anticoagulant, and antioxidant activities using chemical methods rather than intracellular studies [15].

Indomethacin (Ind) is one of the most commonly prescribed nonsteroidal anti-inflammatory drugs to reduce inflammation. Dabigatron (Dab) is a novel oral anticoagulant with an advantage over other anticoagulants, such as warfarin, which can interact with food and drug metabolism [16]. One of the mechanisms of drug-derived cell injury has been reported to involve both uncoupling of mitochondrial oxidative phosphorylation and inhibition of the electron transfer chain, resulting in depletion of intracellular ATP and generation of reactive oxygen species. However, Ind and Dab remain unknown. Therefore, we investigated whether mitochondria-derived ROS is one of the mechanisms of injury induced by these agents. Ind treatment produced superoxide production in mitochondria, and then it subsequently induced lipid peroxide and cellular injury in gastric epithelial cells. In combination with lansoprazole, which is a proton pump inhibitor, intracellular ROS concentration was decreased and attenuated the cell injury [17]. Dab induced mitochondrial ROS production and lipid peroxidation. Antioxidants such as ascorbic acid can help decrease mitochondrial ROS and attenuate the cytotoxicity of Dab [18]. Previously, we reported that cytotoxicity derived from Ind and Dab is induced by an increased mitochondrial ROS level and can be attenuated through the use of antioxidants [17,18]. β-1,3/1,6-glucans, especially laminaran derived from seaweeds, are known to exhibit various bioactive functions; however, in vitro studies on the ROS-scavenging effect of laminaran and its effect on attenuation of cytotoxicity induced by clinical drugs have not yet been conducted. Therefore, we aimed to monitor laminaran activity against ROS using electron spin resonance (ESR), which is a powerful tool for monitoring ROS scavenging. Intracellular ROS levels were also monitored with and without Ind and Dab treatment to study the attenuation of cytotoxicity. It was revealed for the first time that laminaran can scavenge ^1^O_2_ as well as O_2_^•^^−^; significantly suppress production of intracellular radicals such as O_2_^•^^−^, ^•^OH, and ONOO; and attenuate the cytotoxicity caused by commonly prescribed drugs such as Ind and Dab.

## 2. Materials and Methods

### 2.1. Materials

Laminaran, extracted from seaweed *Eisenia bicyclis*, was purchased from Tokyo Chemical Industry (CAS No.: 9008-22-4, Tokyo, Japan). Ind was purchased from Wako Pure Chemical Industries (Osaka, Japan) and dissolved in dimethyl sulfoxide (DMSO) at a concentration of 100 mM and prepared for each experiment. Dab was purchased from Cayman Chemical Co. (Ann Arbor, MI, USA) and dissolved in DMSO at a concentration of 10 mM and stored at −20 °C until use. Acid red and 1-oxyl-2,2,6,6-tetramethyl-4-hydroxypiperidine (TEMPOL) were purchased from Tokyo Chemical Industry (Tokyo, Japan). Hydrogen peroxide (H_2_O_2_) and xanthine were purchased from Wako Pure Chemical Industries. 5-(2,2-dimethyl-1,3-propoxycyclophosphoryl)-5-methyl-1-pyrroline N-oxide (CYPMPO) was purchased from Radical Research Inc. (Tokyo, Japan). Xanthine oxidase was purchased from Nacalai Tesque (Kyoto, Japan).

### 2.2. Electron Spin Resonance Spectroscopy

The ROS-scavenging ability of laminaran was measured via ESR. Laminaran was dissolved in Milli-Q water at a concentration of 500 mg mL^−1^. ROS levels were measured according to the method of Oowada et al. [19]; ^1^O_2_ was generated in combination with acid red and light. This reaction mixture comprised Milli-Q water with 200 µM acid red and 10 mM TEMPOL with or without 50 mg mL^−1^ laminaran. ^•^OH was generated in combination with H_2_O_2_ and light. This reaction mixture comprised Milli-Q water with 10 mM H_2_O_2_ and 10 mM CYPMPO with or without 50 mg mL^−1^ laminaran. O_2_^•^^−^ was generated from a xanthine/xanthine oxidase reaction. The reaction mixture comprised Milli-Q water with 20 mM hypoxanthine, 20 units mL^−1^ xanthine oxidase, and 10 mM CYPMPO with or without 50 mg mL^−1^ laminaran. The ESR spectra of the laminaran-containing mixtures were recorded using a JEOL-TE X-band spectrometer (JEOL, Tokyo, Japan) and compared with those of each mixture without laminaran. ESR spectra were obtained under the following conditions: 20 mW incident microwave power, 9.2 GHz frequency, 0.2 mT modulation width, 7.5 mT sweep width, 0.1 s time contrast, and 335.5 mT center field. All experiments were repeated three times.

### 2.3. Cell Culture

The rat gastric epithelial cell line RGM1 was purchased from the Riken Cell Bank (Ibaraki, Japan). RGM1 cells were cultured in Dulbecco’s modified Eagle medium/F12 supplemented with L-glutamine (Life Technologies Japan Ltd., Tokyo, Japan). The culture medium contained 10% inactivated fetal bovine serum (GE Healthcare Life Sciences, Amersham, UK) and 1% penicillin/streptomycin (Wako Pure Chemical Industries). Cells were cultured in a CO_2_ incubator with 5% CO_2_ at 37 °C.

### 2.4. Measurement of Mitochondrion-Derived ROS

MitoSOX (Thermo Fisher Scientific, Waltham, MA, USA) was used as a fluorescent dye to detect mitochondrial O_2_^•^^−^ in live cells [20]. RGM1 cells were cultured in 24-well plates at a density of 1 × 10^4^ cells well^−1^ and incubated for 2 d. After aspiration of the supernatant, RGM1 cells were incubated in medium containing 0, 1, 10, or 100 µg mL^−1^ laminaran with 1 mM Ind or 50 µM Dab at 37 °C for 1 h. After aspiration of the supernatant, cells were incubated with 5 μM MitoSOX in MSF buffer for 10 min. MSF buffer contains 5.4 mM KCl, 136.9 mM NaCl, 8.3 mM glucose, 0.44 mM KH_2_PO_4_, 0.33 Na_2_HPO_4_, 10.1 mM HEPES, 1 mM MgCl_6_∙6H_2_O, and 1 mM CaCl_2_∙2H_2_O. The intracellular fluorescence intensity of the cells in each treatment group was determined using a fluorescence microscope (IX83; Olympus, Tokyo, Japan). MitoSOX was excited using a 535–555 nm filter and the emission was collected using a 570–625 nm filter.

### 2.5. Detection of the Generation of Intracellular ^•^OH and ONOO^−^

2-[6-(4″-hydroxy) phenoxy-3H-xanthen-3-on-9-yl] benzoic acid (HPF) (Goryo Chemical, Hokkaido, Japan) was used as a fluorescence dye to detect the generation of the intracellular ^•^OH and ONOO^−^ [21]. RGM1 cells were cultured in 24-well plates at a density of 1 × 10^4^ cells well-1 and incubated for 2 days. After aspiration of the supernatant, RGM1 cells were incubated in medium containing 0, 1, 10, or 100 µg mL^−1^ laminaran with 1 mM Ind or 250 µM Dab at 37 °C for 1 h. After aspirating the supernatant, the cells were incubated with 5 μM HPF in MSF buffer medium for 15 min to incorporate HPF into the cells, and the medium was replaced with MSF buffer medium to increase the signal-to-noise ratio. The fluorescence intensity of the cells in each treatment group was determined using a fluorescence microscope (Olympus, IX83). HPF was excited using a 460–495 nm filter and the emission was collected using a 510–550 nm filter. 

### 2.6. Cell Viability Assay

Cell viability was evaluated using the Cell Counting Kit-8 (CCK8) (Dojindo, Tokyo, Japan). The cells were cultured in 96-well plates at a density of 5 × 10^3^ cells well^−1^ and incubated for 2 d. The supernatant was aspirated, and the cells were cultured in a medium containing 0, 1, 10, and 100 µg mL^−1^ laminaran with 300 µM Ind or 15 µM Dab for 24 h. After cultivation, the cells were incubated within 10% CCK8. As CCK-8Absorbance at 450 nm was measured using a Synergy H1 microplate reader (BioTek Instruments Inc., Winooski, VT, USA). 

### 2.7. Statistical Analysis

All of the experiments of mitochondrion-derived ROS, intracellular ^•^OH and ONOO^−^, and cell viability assay were conducted in triplicates. Data are expressed as the mean ± standard deviation and were assessed using analysis of variance. Individual groups were compared using Tukey’s post hoc or Student’s *t*-test with *p* < 0.05 and *p* < 0.01 considered significant.

## 3. Results

### 3.1. ROS-Scavenging Effect of Laminaran Extract

**Figure 1:**
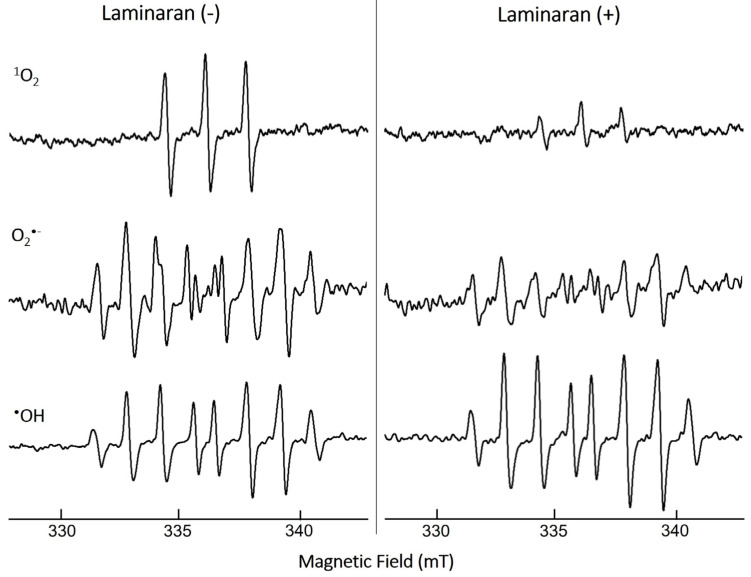
ESR spectra of ^1^O_2_, O_2_^•^^−^, and ^•^OH spin adducts with (+) and without (−) laminaran and their scavenging activity.

The ROS-scavenging ability of laminaran was evaluated via ESR spectroscopy under cell-free conditions through measuring the spin adduct signal. We analyzed three different free radicals: ^1^O_2_, O_2_^•^^−^, and ^•^OH (Figure 1). The quenching ability of laminaran for the corresponding free radical was monitored based on the change in peak height. The signal peaks of ^1^O_2_ and O_2_^•^^−^ were lower for laminaran than for the control, indicating the removal of these ROS by laminaran. The peak height of ^•^OH signal was higher than that of the control, indicating that laminaran could not scavenge ^•^OH (Figure 1). From the ESR spectra, it was found that laminaran could scavenge ^1^O_2_ and O_2_^•^^−^. All of the experiments of mitochondrion-derived ROS, intracellular ^•^OH and ONOO^−^, and cell viability assay were conducted in triplicates. Data are expressed as the mean ± standard deviation and were assessed using analysis of variance. Individual groups were compared using Tukey’s post hoc or Student’s *t*-test with *p* < 0.05 and *p* < 0.01 considered significant.

### 3.2. Scavenging Effect of Laminaran on Mitochondrial ROS Induced by Ind and Dab Treatment

**Figure 2:**
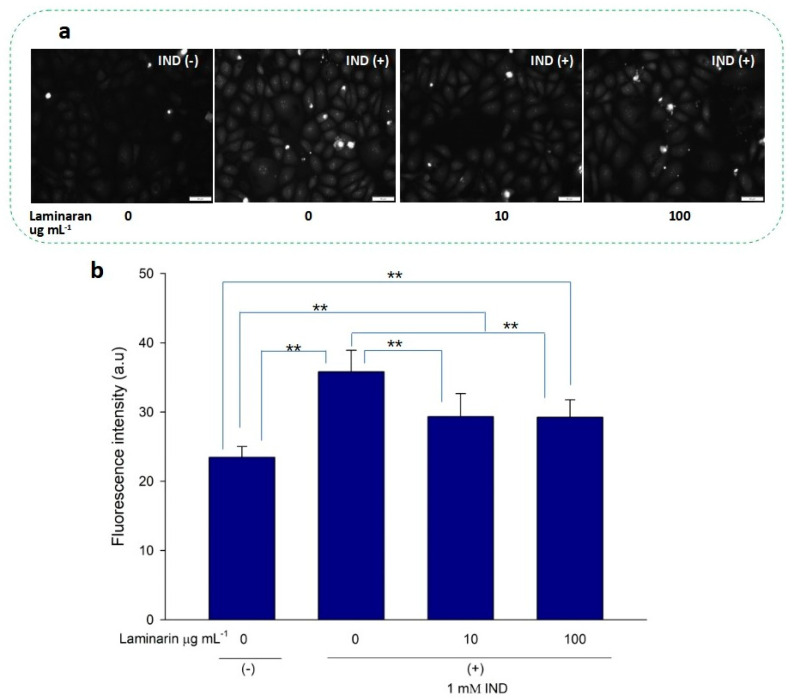
(**a**) Fluorescence microscopic image of RGM1 cells with mitoSOX dye after IND exposure with and without laminaran treatment. Scale bar, 50 µm. (**b**) Corresponding relative fluorescence intensity per cell represented as arbitrary units. Error bars represent means ± SD (*n* = 10). Excitation wavelength = 535–555 nm and emission wavelength = 570–625 nm. ** *p* < 0.01.

**Figure 3:**
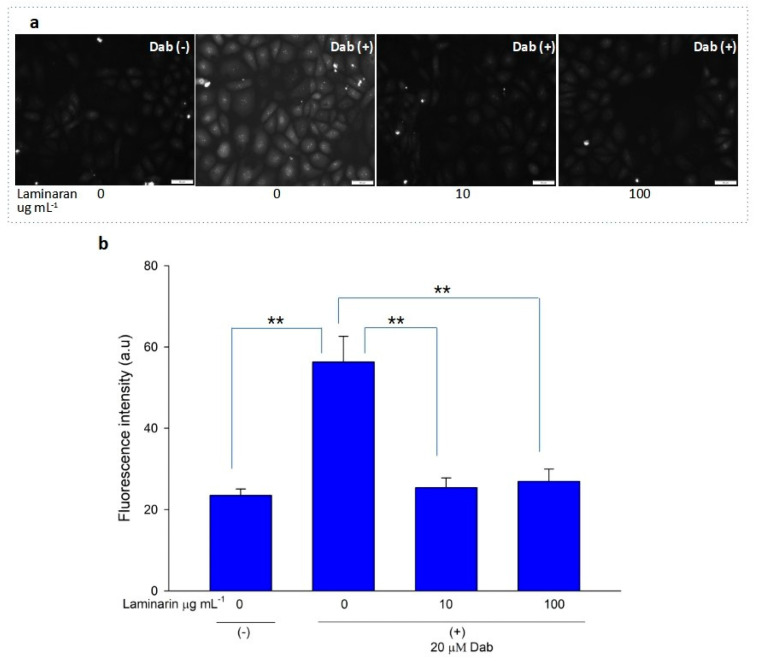
(**a**) Fluorescence microscopic image of RGM1 cells treated with mitoSOX dye after Dab exposure with and without laminaran treatment. Scale bar, 50 µm. (**b**) Corresponding relative fluorescence intensity per cell represented as arbitrary units. Error bars represent the mean ± standard deviation (*n* = 10). Excitation wavelength = 535–555 nm and emission wavelength = 570–625 nm. ** *p* < 0.01.

Free-radical-mediated cytotoxicity is relatively more distinct in mitochondrial cells. Therefore, we analyzed the effect of laminaran on O_2_^•^^−^ induction by autoinflammatory drugs using the mitochondrial ROS dye MitoSOX as a marker. MitoSOX contains dihydroethidium. When dihydroethidium is oxidized by O_2_^•^^−^, it is converted to 2-hydroxyethidium, thereby increasing intracellular fluorescence intensity in a dose-dependent manner with mitochondrion-derived O_2_^•−^ [22]. We artificially induced ROS production in RGM1 cells using 1 mM Ind or 25 µM Dab. MitoSOX fluorescence analysis showed that Ind-treated cells showed a higher fluorescence intensity than that of untreated cells, indicating the induction of ROS production by Ind (Figure 2a). When Ind-treated cells were incubated with 10 and 100 µg mL^−1^ laminaran, there was a significant decrease in fluorescence intensity (*p* < 0.01) (Figure 2b). A similar increase in fluorescence intensity was observed in the Dab-treated RGM1 cells, indicating enhanced ROS production (Figure 3a). After laminaran treatment, ROS-producing cells showed a rapid decrease in the fluorescence signal from MitoSOX (Figure 3b). Notably, laminaran had a greater effect on Dab-treated cells than on Ind-treated cells. It was estimated that laminaran could scavenge mitochondrial O_2_^•^^−^ induced by the administration of Ind and Dab by up to 16.64% and 54.80%, respectively. From mitochondrial O_2_^•^^−^ marker dye analysis, we confirmed that laminaran, at both high (100 µg mL^−1^) and low (10 µg mL^−1^) concentrations, could efficiently attenuate drug-induced O_2_^•^^−^ production.

### 3.3. Supression Effect of Laminaran on Intracellular ^•^OH and ONOO^−^

**Figure 4:**
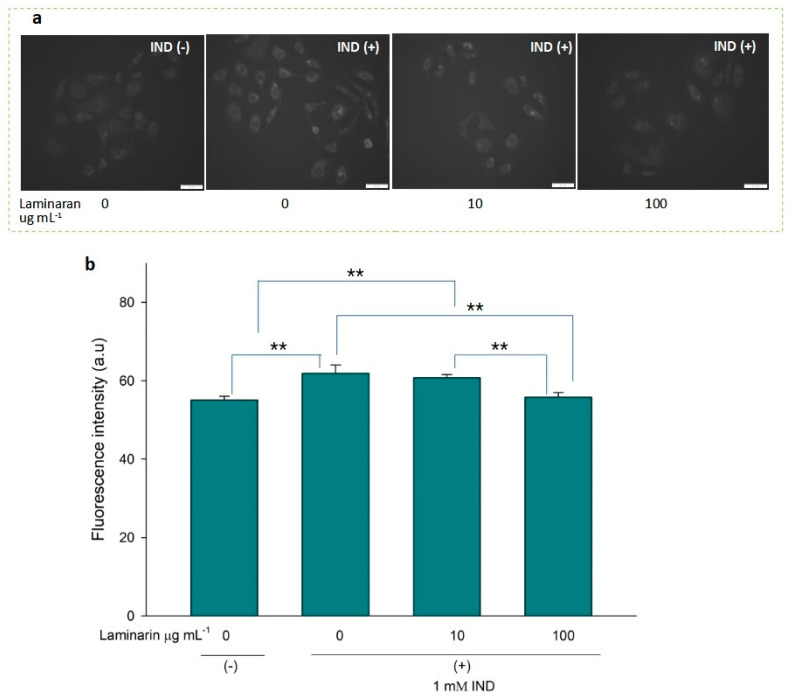
(**a**) Fluorescence microscopic image of RGM1 cells treated with HPF after IND exposure under different concentrations of laminaran treatment. Scale bar, 50 µm. (**b**) Corresponding fluorescent intensity per cell represented as arbitrary units. Error bars represent the mean ± standard deviation (*n* = 10). Excitation wavelength = 460–495 nm and emission wavelength = 510–550 nm. ** *p* < 0.01.

**Figure 5:**
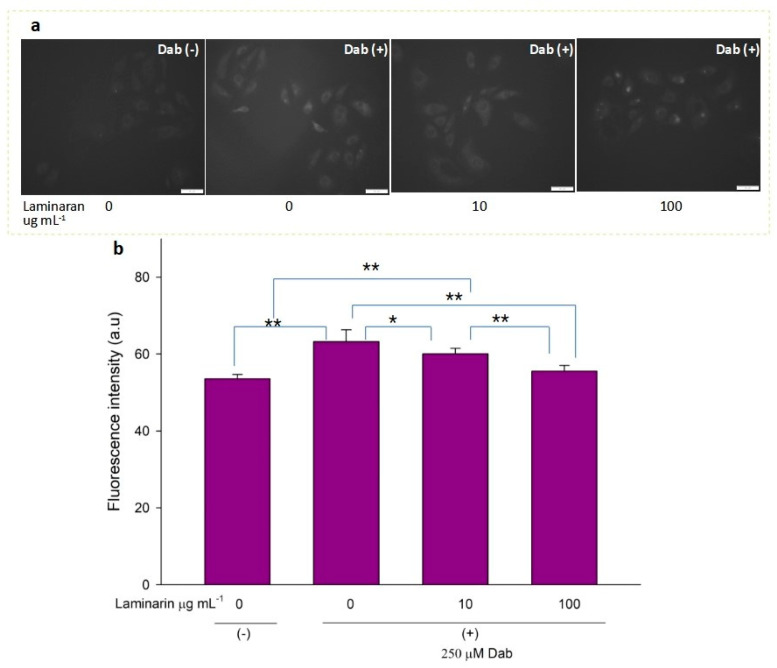
(**a**) Fluorescence microscopic image of RGM1 cells treated with HPF after Dab exposure under different concentrations of laminaran treatment. Scale bar, 50 µm. (**b**) Corresponding fluorescence intensity per cell represented as arbitrary units. Error bars represent the mean ± standard deviation (*n* = 10). Excitation wavelength = 460–495 nm and emission wavelength = 510–550 nm. * *p* < 0.05, ** *p* < 0.01.

^•^OH and ONOO^−^ actively interfere with many biological processes, leading to apoptosis. ESR analysis demonstrated that laminaran did not show a significant scavenging effect on ^•^OH, but only on oxygenic free radicals (Figure 1). However, MitoSox fluorescence analysis showed that laminaran treatment attenuated drug-induced mitochondrial O_2_^•−^ production. It is known that O_2_^•^^−^ is intracellularly converted into ^•^OH and H_2_O_2_. Therefore, to investigate whether ^•^OH and ONOO^−^ production could be suppressed via attenuation of drug-induced mitochondrial O_2_^•^^−^ production by means of laminaran treatment, we used a novel highly specific fluorescent probe, HPF. HPF itself does not emit any florescence; however, when it strongly reacts with ^•^OH and ONOO^−^, it emits strong fluorescence in a dose-dependent manner. Similar to our earlier experiments using MitoSOX, we observed a significant increase (*p* < 0.01) in the fluorescence intensity of both Ind- and Dab-treated RGM1 cells after incorporating HPF. This increase in fluorescence indicated that both drugs induced intracellular ^•^OH and ONOO^−^ production (Figure 4a and Figure 5b). When laminaran was added at a concentration of 100 µg mL^−1^ to Ind treated cells (Figure 4b) and Dab-treated cells (Figure 5b), significant reductions (*p* < 0.01) were observed in the HPF fluorescence signals at the concentration of 100 µg mL^−1^. Further, a less significant reduction (*p* < 0.05) was observed when laminaran was administered at a concentration of 10 µg mL^−1^ to Dab-treated cells (Figure 5b). These results suggest that laminaran indirectly suppresses drug-derived mitochondrial ^•^OH and ONOO^−^ production through attenuating mitochondrial O_2_^•^^−^ production.

### 3.4. Effect of Laminaran on Drug-Induced Cytotoxicity

**Figure 6:**
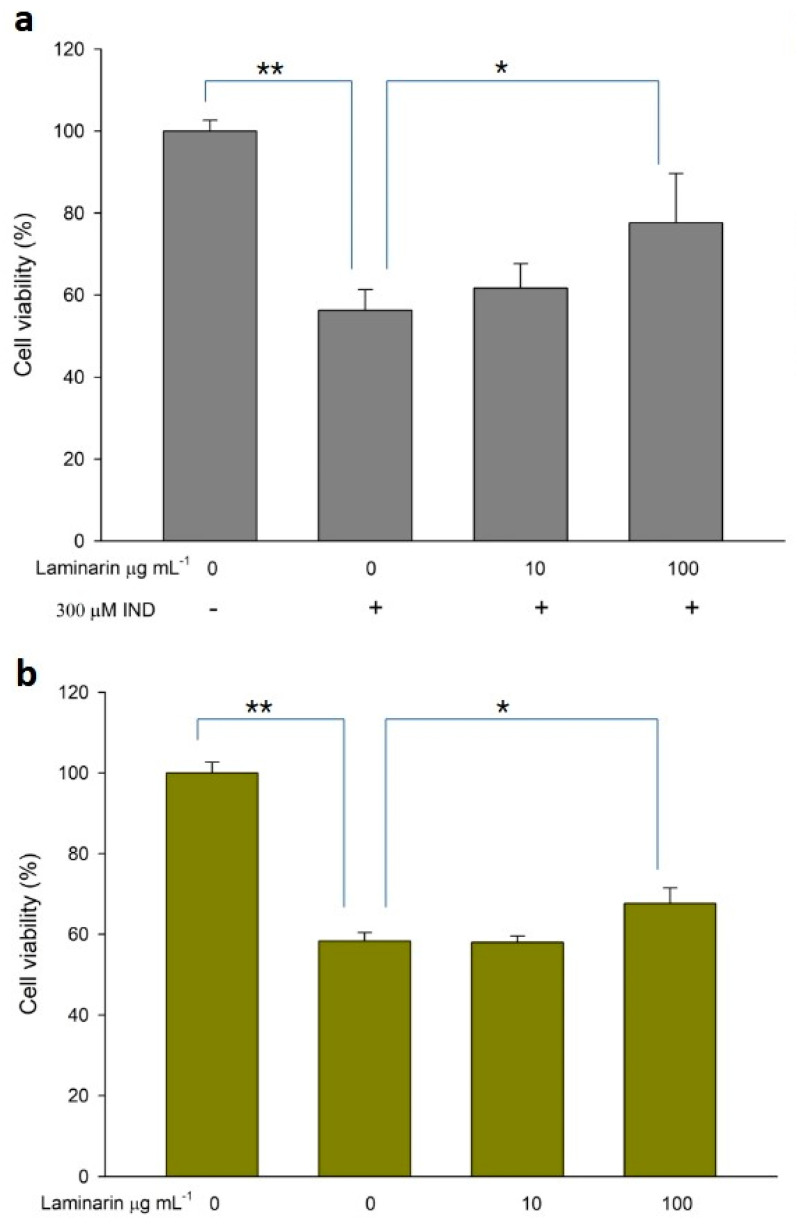
Cytotoxicity of (**a**) IND and (**b**) Dab. Error bars represent the mean ± standard deviation (*n* = 4). * *p* < 0.05, ** *p* < 0.01.

ROS production induced by drug toxicity can cause cellular damage, leading to cell death; antioxidant treatment can alleviate this toxicity. Therefore, we evaluated the effect of laminaran on cell death in WST cell viability assays. When RGM1 cells were incubated with Ind (300 µM) or Dab (15 µM) in the presence of laminaran at two different concentrations (10 and 100 mg m L^−1^), we observed significant differences in cell viability (Figure 6). Ind treatment resulted in a 43.7% reduction in cell viability compared with that in cell cultures without Ind. Laminaran treatment restored cell viability in a dose-dependent manner with 100 µg mL−1 treatment resulting in cell viability up to 77.6 ± 12% (Figure 6a). The viability of cells treated with Dab was 58.3 ± 2.1% compared with that of the control (100%). Laminaran at 100 µg mL^−1^ partially recovered cell viability by 8.7%, but not at 10 µg mL^−1^ (Figure 6b). 

## 4. Discussion

Kelps have been traditionally consumed mainly as food and condiments in East Asian countries and are known to produce a β-1,3/1,6-glucan called laminaran. This study investigated whether laminaran could attenuate the cytotoxicity caused by clinical drugs including Ind and Dab, which are used worldwide as an anti-inflammatory and anti-coagulant, respectively, because cytotoxicity is a major problem that needs to be addressed in the clinical field.

We first performed ESR spectroscopy, a highly sensitive method for free-radical detection, via which radical concentrations as low as 10–12 M can be detected. In ESR analysis, when radicals react with a spin-trapping agent, spin adduct radicals are produced, which enables the identification of ROS and the amount of free radicals based on the signal shape and intensity of the spin adduct radicals [23]. O_2_^•^^−^ is the precursor of most ROS such as ^•^OH and enhances oxidative chain reactions. The signals of ^1^O_2_ and O_2_^•^^−^ were significantly decreased in laminaran treatment compared with those of the control (non-laminaran treatment), indicating that laminaran has an antioxidant effect that scavenges ROS of ^1^O_2_ and O_2_^•^^−^ (Figure 1). This result is consistent with previous studies reporting that laminaran exerts an antioxidant effect against O_2_^•^^−^ [24]. However, it was demonstrated for the first time that laminaran can also scavenge ^1^O_2_. It is generally believed that intracellular O_2_^•^^−^ is scavenged by MnSOD and ^•^OH is scavenged by catalase; however, ^1^O_2_ cannot be scavenged by antioxidant enzymes. Intracellular ^1^O_2_ is generated via both light-dependent and light-independent mechanisms. Compared to other ROS such as O_2_^•^^−^ and ^•^OH, there is no antioxidant enzyme that can scavenge ^1^O_2_. Carotenoids are organic pigments that are naturally produced by plants, algae, bacteria, and fungi, and they are reported to scavenge ^1^O_2_ strongly. These compounds can prevent not only photoaging effects [25] but also reduce the concentration of plasma lipid peroxidation [26]. Tomatoes, which contain lycopene, can especially decrease LDL oxidizability and 8-iso-PGF2α. These results demonstrated that tomato product consumption can prevent lipid peroxidation, a risk factor of atherosclerosis and cardiovascular disease [27]. Similarly, our study demonstrated for the first time the ^1^O_2_ scavenging effect of seaweed laminaran, and our results also support the claim that regular consumption of kelp can help prevent ^1^O_2_ derived diseases.

We evaluated the antioxidant effects of laminaran on intracellular mitochondrion-derived O_2_^•^^−^ in vitro. Adenosine triphosphate (ATP) is the main energy source in mammalian cells and is produced via oxidative phosphorylation in the mitochondria. To produce ATP, mitochondria use a series of protein complexes located on the inner membrane. These complexes transport electrons and sequentially translocate protons into the intermembrane space. These processes create a proton gradient that is essential for ATP generation [28]. Following oxidative phosphorylation, O_2_^•^^−^ is generated in the mitochondria and converted to other ROS, such as ^•^OH and H_2_O_2_; thus, elimination of mitochondrial O_2_^•^^−^ is essential for efficient cellular functioning. Previously, we reported that Ind and Dab enhance ROS production in mitochondrial cells [18]. Staining using MitoSOX, a specific O_2_^•^^−^ dye, demonstrated that laminaran could facilitate scavenging intracellular O_2_^•^^−^ produced via IND and Dab treatment (Figure 2 and Figure 3). 

We further investigated the effect of laminaran on the generation of ^•^OH and ONOO^−^ using HPF. O_2_^•^^−^ is degraded by superoxide dismutase and converted to H_2_O_2_. ^•^OH is generated from the Fenton reaction between ferrous iron and H_2_O_2_ [29]. Moreover, O_2_^•^ reacts with nitric oxide in aqueous solutions to generate ONOO [30]. In the present study, ESR analysis showed that laminaran could not scavenge ^•^OH but scavenges ^1^O_2_ and O_2_^•^^−^ (Figure 1). Therefore, we investigated whether laminaran can suppress the generation of ^•^OH and ONOO via the removal of O_2_^•^^−^. The intracellular fluorescence intensity of HPF in cells exposed to Ind or Dab with laminaran treatment was significantly lower than that in cells without laminaran (Figure 4 and Figure 5). Therefore, it was suggested that laminaran cannot scavenge ^•^OH directly; however, it can scavenge ^•^OH and ONOO indirectly via suppression of the generation of these radicals caused by O_2_^•^^−^ scavenging.

We evaluated whether laminaran could attenuate cytotoxicity induced by Ind or Dab treatment. These drugs induce side effects, such as gastrointestinal bleeding or esophageal disorders, in clinical cases [31,32]. In particular, as Ind is combined with anti-ulcer drugs to protect the gastric mucosa, reduction of Ind-derived side effects is an important issue in the clinical field. We found that the viability of Ind-treated cells was significantly decreased compared with that of non-Ind-treated cells, whereas in cells treated with Ind and 100 µg mL^−1^ laminaran, cell viability was increased significantly compared with that of cells treated without laminaran. A similar recovery trend of cell viability was obtained using Dab treatment; however, the recovery rate was lower than that observed for Ind. From these results, it was concluded that laminaran can significantly attenuate the cytotoxicity caused by Ind and, to a lesser extent, that caused by Dab. Laminaran has the potential to be used as a protective agent.

A few studies have reported that laminaran has anticancer and anti-inflammatory activity, immunostimulatory activity, and potential anti-aging effects [33]. In particular, the latter authors reported that laminaran from *Laminaria digitata* decreases intracellular ROS level significantly from the low concentration of 1 µg mL^−1^ in adult human dermal fibroblasts. In skin aging, ROS secretion can lead to various cellular damages, suggesting that laminaran is a promising functional food with potential anti-aging effects. In the present study, we also revealed that laminaran scavenges intracellular O_2_^•^^−^ and suppresses the generation of ^•^OH and ONOO which induces DNA damage or lipid peroxidation, consequently causing many diseases, such as cancer, cardiovascular disease, and neurological disease. Therefore, we expect that laminaran may have a preventive effect against these diseases and have anti-aging effects. These effects should be investigated in vivo in future studies. Authors should discuss the results and how they can be interpreted from the perspective of previous studies and of the working hypotheses. The findings and their implications should be discussed in the broadest context possible. Future research directions may also be highlighted.

## 5. Conclusions

Using ESR assay, we determined that laminaran can scavenge two types of ROS, ^1^O_2_ and O_2_^•^^−^, directly, but it does not scavenge ^•^OH. Mitochondrial ROS detection dye showed that laminaran scavenged intracellular mitochondrial O_2_^•^^−^ produced upon administration of Ind or Dab. Moreover, significant reductions in ^•^OH and peroxynitrate ONOO levels were observed. Since ^•^OH and ONOO are generated from O_2_^•^^−^ in the cells, laminaran could indirectly suppress the generation of ^•^OH and ONOO via the removal of O_2_^•^^−^. Both Ind and Dab induce cell injury via ROS production. Laminaran attenuated the cytotoxicity derived from these drugs.

## Data Availability

The data sets generated during/or analyzed during the current study are available from the corresponding author on reasonable request.
